# Supplementary material for: Deep‐learning based fully automatic segmentation of the globus pallidus interna and externa using ultra‐high 7 Tesla MRI
Source: Hum Brain Mapp. 2021 Mar 18;42(9):2862–79. doi: 10.1002/hbm.25409 (PMC8127160; doi:10.1002/hbm.25409)
Supplement: Supplementary file 3 — APPENDIX S1: Supporting information [file HBM-42-2862-s003.pdf]

Supplementary Materials

In this supplementary materials, we provide an additional analysis for the MSD metric, obtained by GP-net for the test cohort. The MSD metric provides a single number which characterizes the average surface distance between the segmentations and manual delineations. However, since this number is an average, it does not tell the reader whether there are areas in which there are systematically larger differences. To gain more insight regarding the regions in which larger surface errors occur with GP-net's segmentations, we provide the following analysis. For each subject from the test cohort, we calculated the MSD (with respect to the corresponding ground truth segmentation) at each point along the edges (contours) of the manual delineation. MSD was calculated using the distance transform applied to the GP-segmentations. The values along the contours of the manual segmentations were taken and registered to MNI space to produce the maps presented in Fig. S1 and averaged along the voxel dimension.

Figure S1 shows two slices which are the most relevant for DBS surgery, and focus on the lamina borders between the two structures. The left panel shows an axial slice at the AC level while the right slice shows a more dorsal slice. As shown in the heatmap, the largest differences occur on the lateral and dorsal borders of the GPi. As expected, the areas with the highest MSD are transition points where the lamina is found between the GPi and GPe; this may, in part, be explained due to partial volume and image resolution that characterize the uncertainty of the exact border region. As can be observed, most errors are on the order, or below the resolution of 1.5 times a single voxel in the resampled grid.

We further provide a glimpse to the performance of DL based architectures and GP-net in particular when faced with out-of-distribution (OOD) data. Figure S2 presents dice scores for the segmented GPe and GPi from 1.5 T and 3 T T2 scans (32 subjects, two healthy, 7 diagnosed with ET and the rest diagnosed with PD) acquired using standard clinical protocols and varying resolutions (e.g., 1 or 0.8 mm^3 isotropic resolution, 0.8 mm in-plane resolution and 2 mm slice thickness, or 0.5 mm in-plane resolution and a 2 mm slice thickness). All images were resampled to the 0.39 mm^3 isotropic grid. In this case, we performed inference on the clinical 3 T T2 scans using GP-net, which was trained only with 7 T T2 images. As expected, since the clinical images differ from the trained 7 T T2 images' data (field strength, resolution, contrast) its performance degraded. This is a common limitation in the DL literature and is an active topic of research (Liu et al., 2020). Extending GP-net to handle additional contrasts and resolutions is a matter of future research.

Figure S1. Left panel shows a GPi MSD heatmap from an axial slice along the AC line. Right panel illustrates a GPi MSD heatmap plotted along a more dorsal slice.

Figure S2. GP-net performance with OOD data. Dice scores for GP-net segmentations of the GPe (blue) and GPi (orange) from clinical 3 T T2 scans, with varying image resolutions, when trained on 7 T T2 images only.

References

Liu, W., Owens, J. D., Wang, X., & Li, Y. (2020). Energy-based out-of-distribution detection. *ArXiv:2010.03759*.
